# CD4 and CD8 T Cell Responses to the *M. tuberculosis* Ag85B-TB10.4 Promoted by Adjuvanted Subunit, Adenovector or Heterologous Prime Boost Vaccination

**DOI:** 10.1371/journal.pone.0005139

**Published:** 2009-04-09

**Authors:** Tara Elvang, Jan P. Christensen, Rolf Billeskov, Truc Thi Kim Thanh Hoang, Peter Holst, Allan Randrup Thomsen, Peter Andersen, Jes Dietrich

**Affiliations:** 1 Department of Infectious Disease Immunology, Statens Serum Institute, Copenhagen, Denmark; 2 Institute of Medical Microbiology and Immunology, the Panum Institute, Copenhagen, Denmark; Universita di Sassari, Italy

## Abstract

**Background:**

Although CD4 T cells are crucial for defense against *M.tb,* it is still not clear whether the optimal response against *M.tb* in fact involves both CD4 and CD8 T cells. To test this, we used a new vaccine strategy that generated a strong balanced T cell response consisting of both CD4 and CD8 T cells.

**Methods and Findings:**

To compare CD4 and CD8 responses against Ag85B-TB10.4 (H4), H4 was delivered as a subunit vaccine in cationic liposomes (CAF01), expressed in Ad5 (Ad-H4) or as a heterologous prime boost vaccination. H4/CAF01 induced primarily CD4 T cells and Ad-H4 gave predominantly a CD8 T cell response. In contrast, the heterologous prime boost combination resulted in augmentation of both the CD4 and CD8 response. The majority (>40%) of the CD4 T cells induced by the heterologous prime boost protocol were polyfunctional, and expressed IFN-γ^+^, IL-2^+^, and TNF-α^+^, whereas most of the CD8 T cells expressed IFN-γ^+^ and TNF-α^+^ and possessed strong cytotoxic potential. The heterologous prime boost protocol also gave an increase in protective efficacy against *M.tb* challenge compared to H4/CAF01 and Ad-H4. Both the H4 specific CD4 and CD8 T cells were recruited to the site of infection, at the onset of infection. However, compared to CD8 T cells, CD4 T cells showed more extensive recruitment and were the main T cell subset proliferating at the site of infection.

**Conclusions/Significance:**

Heterologous prime boost based on H4, produced an additive effect on the priming of CD4 and CD8 cells and in terms of the protective capacity of the vaccine, and therefore represent an interesting new vaccine strategy against *M.tb*. However, CD4 and CD8 T cells respond very differently to live *M.tb* challenge, in a manner which supports the consensus that CD4 T cells do play the major role during the early stages of an *M.tb* infection.

## Introduction

Approximately one-third of the world's population is latently infected with *Mycobacterium tuberculosis (M.tb)*, and understanding the immune mechanisms involved in controlling this chronic disease is of high priority. The major mechanisms of cell-mediated immunity include CD4 Th1-cell mediated activation of macrophages to destroy intracellular bacterial pathogens, and the central role of IFN-γ in the control of tuberculosis has been clearly demonstrated by the susceptibility to mycobacterial infections in mice with a disrupted IFN-γ gene and in humans with mutations in genes involved in the IFN-γ and IL-12 pathways [1]–[4].Unlike CD4 T cells, which are crucial in the defence against *M.tb*, the role of CD8 T cells is not fully resolved. However, numerous studies have indicated that cytotoxic CD8 T cell-mediated killing of infected host cells do play a role in the defence against a *M.tb* infection [5]–[10]. Other results in favour of the protective effect of CD8 T cells come from recent studies, showing that lack of the RD-1 region in BCG (compared to *M.tb*) is responsible for the reduced CD8 T cell response induced by BCG compared to *M.tb*, and that re-introducing RD-1 into BCG selectively restored the CD8 T cell response and importantly also increased the protective efficacy of the vaccine [11]–[14]. As a consequence there has recently been increased focus on the CD8 T cells and several new vaccine strategies that target these T cells are currently pursued in many laboratories involved in TB vaccine research. Viral vectors, such as adeno- or vaccinia virus, trigger a Th1-dominated immune response, characterized by potent induction of IFN-γ, and are also efficient in inducing a robust CD8 T cell response against their target antigens. One *M.tb* vaccine of this type is MVA-85A, a replication-deficient recombinant vaccinia virus, expressing antigen 85A from *M.tb*. This vaccine has performed well in animal models where it induces not only CD8 T cells, but also CD4 T cells, and significant protection against *M.tb*. Results from clinical trials indicate that it is also immunogenic in humans but with a response almost exclusively composed of CD4 cells [15], [16]. Replication-deficient recombinant adenovirus for comparison are widely accepted as one of the most efficient delivery platforms for the induction of a CD8 T cell dominated response and adeno vectors have been evaluated in a number of studies of TB antigens. The H1 molecule (Ag85B-ESAT-6) expressed in Ad5 was demonstrated to promote a strong response consisting exclusively of CD8 cells directed to a few immunodominant epitopes but unable to protect against TB [17]. Another virally-vectored vaccine is Aeras-402 Ad35, expressing a fusion of antigen 85A, antigen 85B, and TB10.4. All three antigens are present in *M.tb* and BCG, and all are highly immunogenic. The characterization of this vaccine in animal models has so far been limited but also this vaccine is clearly capable of inducing a strong CD8 T cell responses [18].

The antigens Ag85B and TB10.4 have been extensively characterized [11], [19]–[22] and a fusion protein consisting of both Ag85B-TB10.4 adjuvanted with cationic liposomes induced a strong protection against infection with *M.tb* [23]. In the present study we have compared CD4 and CD8 responses against the H4 molecule delivered as a subunit vaccine in cationic liposomes (CAF01), expressed in Ad5 (Ad-H4) or as a heterologous prime boost vaccination. Our data demonstrate that the heterologous prime boost vaccination gives higher levels of both polyfunctional CD4 T cells, cytotoxic CD8 T cells and protection against *M.tb* challenge than Ad-H4 or rH4 on their own. When we monitored cellular infiltrate in the lung post *M.tb* challenge in mice vaccinated by the heterologous prime boost protocol we observed that whereas high numbers of both H4 specific CD4 and CD8 T cells were promoted by the vaccine, the CD4 T cells showed accelerated recruitment to the site of infection compared to CD8 cells and importantly CD4 cells were the main subset proliferating in the lung in response to *M.tb* infection in the vaccinated animals.

## Results

### Induction and characterization of the vaccine induced antigen specific T cell responses

**Figure 1:**
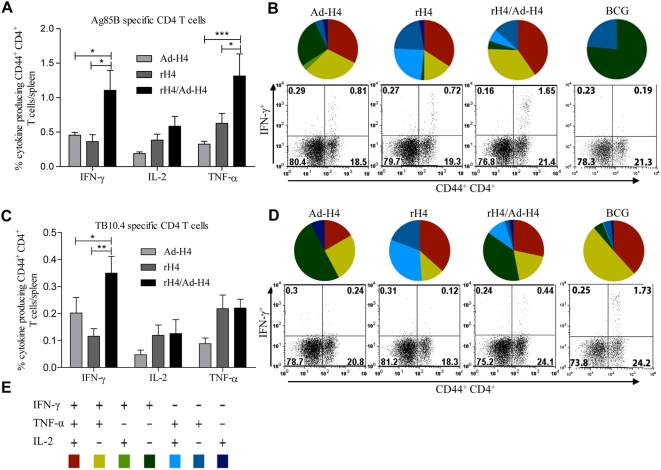
Cytokine frequencies and phenotypic profiles of specific CD4 T cells in Ad-H4, rH4, or rH4/Ad-H4 vaccinated mice one week after final vaccination. CB6F1 mice were vaccinated once with Ad-H4, twice with rH4 in cationic liposomes, or once with rH4 in cationic liposomes followed two weeks later by one Ad-H4 vaccination. Mice were sacrificed one week after the final vaccination and splenocytes were stimulated with Ag85B or TB10.4 peptides prior to staining with anti-CD4, -CD44, -IFN-γ, -IL-2, and –TNF-α. (A and C) Frequencies represent IFN-γ, IL-2, or TNF-α producing CD44^+^CD4^+^ T cells out of total CD4 T cells specific for Ag85B (A) or TB10.4 (C). Background staining from cells stimulated with medium alone has been subtracted. Data represent the mean+SEM of a minimum of five mice per group with ***, p<0.001, **, p<0.01, and *, p<0.05 (2-way ANOVA with Bonferroni posttest). (B and D) IFN-γ, IL-2, and TNF-α cytokine profiles of Ag85B (B) or TB10.4 (D) specific CD4 T cells shown as triple, double, or single positive CD4 T cells. The color code for the pies is shown in (E). Dot plots representative of five mice are also shown in (B) and (D), depicting IFN-γ expressed by CD4^+^CD44^+^ cells after stimulation with Ag85B (B) or TB10.4 (D).

**Figure 2:**
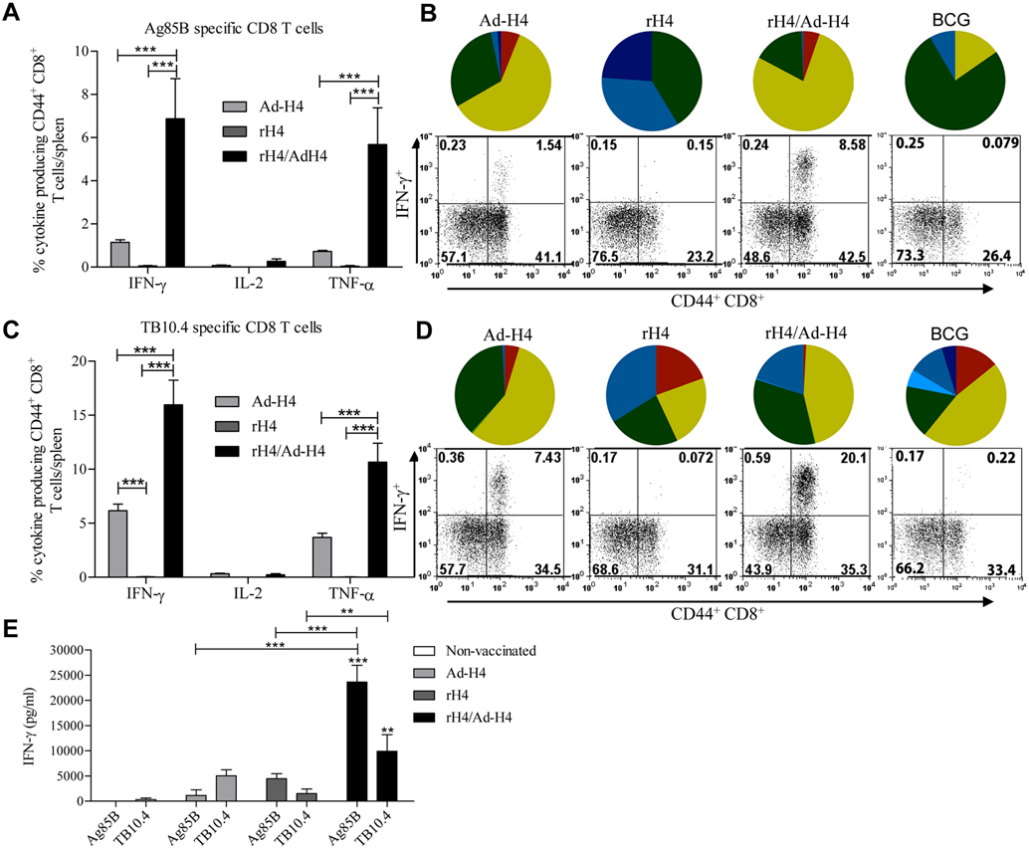
Cytokine frequencies and phenotypic profiles of specific CD8 T cells in Ad-H4, rH4, or rH4/Ad-H4 vaccinated mice one week after final vaccination. CB6F1 mice were vaccinated once with Ad-H4, twice with rH4 in cationic liposomes, or once with rH4 in cationic liposomes followed two weeks later by one Ad-H4 vaccination. Mice were sacrificed one week after the final vaccination and splenocytes were stimulated with Ag85B or TB10.4 peptides prior to staining with anti-CD8, -CD44, -IFN-γ, -IL-2, and –TNF-α. (A and C) Frequencies represent IFN-γ, IL-2, or TNF-α producing CD44^+^CD8^+^ T cells out of total CD8 T cells specific for Ag85B (A) or TB10.4 (C). Background staining from cells stimulated with medium alone has been subtracted. Data represent the mean+SEM of a minimum of five mice per group with ***, p<0.001, **, p<0.01, and *, p<0.05 (2-way ANOVA with Bonferroni posttest). (B and D) IFN-γ, IL-2, and TNF-α cytokine profiles of Ag85B (B) or TB10.4 (D) specific CD8 T cells shown as triple, double, or single positive CD8 T cells. The color code for the pies is the same as shown in Fig. 1E. Dot plots, representative of five mice, are also shown in (B) and (D), depicting IFN-γ expressed by CD44^+^CD8^+^ cells after stimulation with Ag85B (B) or TB10.4 (D). (E) Cells were stimulated with Ag85B or TB10.4 in vitro for 72 hours and IFN-γ levels in supernatants were assessed by ELISA. Data represent the mean+SEM of a minimum of five mice per group with ***, p<0.001, ** and p<0.01 compared with non-vaccinated group, unless otherwise indicated (2-way ANOVA with Bonferroni posttest).

We first compared the immune response promoted by H4 delivered in cationic liposomes (CAF01) expressed in an adeno5 vector or as a hetrologous pirme boost. In particular, we were interested in whether we could induce both CD4 and CD8 T cells, as well as in the quantity and phenotype of these cells. CB6F1 mice were vaccinated once with rH4 followed by a booster Ad-H4 vaccination. Non-vaccinated mice were also included but had no detectable T cells responses to the vaccine components (data not shown). One week after the last vaccination, mice were sacrificed and splenocytes were stimulated *in vitro* with Ag85B and TB10.4, and the production of IFN-γ, IL-2, and TNF-α by CD4 and CD8 T cells was analyzed by intracellular FACS. We found that all three vaccines, Ad-H4, rH4, and rH4/Ad-H4, elicited Ag85B specific CD4 T cells producing all three cytokines (Fig. 1A) and a significant number of these cells were moreover triple positive (35, 33, and 42%, respectively) (Fig. 1B). However, only rH4 and rH4/Ad-H4 vaccinated mice contained TNF-α^+^IL-2^+^ double positive CD4 T cells (Fig. 1B and D). The triple positive and TNF-α^+^IL-2^+^ double positive T cell phenotypes generally represent effector memory- and central memory T cells, respectively [24], indicating the induction of a more memory like CD4 T cell immune response by the vaccines rH4 and rH4/Ad-H4, as compared to Ad-H4. Most importantly, the highest number of antigen specific T cells was seen in mice vaccinated with rH4/Ad-H4. This vaccine induced a higher frequency of both IFN-γ (1.1%±0.3) and TNF-α (1.3%±0.3) producing Ag85B specific CD4 T cells compared to Ad-H4 and rH4 (p<0.001 and 0.05, respectively) (Fig. 1A), and the phenotypic profile of the vaccine induced T cell response reflected the combination of Ad-H4 and rH4 vaccinations since it contained a high frequency of triple- and double positive memory CD4 T cells, as well as a significant amount of IFN-γ^+^TNF-α^+^ double positive (and single positive IFN-γ^+^) effector CD4 T cells (Fig 1B and D) [24], [25]. In general, the level of TB10.4 specific CD4 T cells was found to be low in all three vaccinated groups, however, with a significant increase of IFN-γ producing CD4 T cells in the rH4/Ad-H4 vaccinated mice (Fig. 1C). In comparison, BCG vaccinated mice showed low numbers of Ag85B specific CD4 T cells, but the highest numbers of TB10.4 specific T cells of which the major phenotypes were IFN-γ^+^TNF-α^+^IL-2^+^ or IFN-γ^+^TNF-α^+^ expressing CD4 T cells. In terms of CD8 T cells, we found that only the mice vaccinated with Ad-H4 or rH4/Ad-H4 had detectable numbers of antigen specific CD8 T cells (Fig. 2A and C), but by combining rH4 and Ad-H4 a larger CD8 T cell population was observed. We observed an increase in Ag85B specific IFN-γ producing CD8 T cells from 1.1%±0.1 in the Ad-H4 group to 6.8%±1.9 in the rH4/Ad-H4 group (p<0.001), and an increase from 6.2%±0.6 to 16%±2.3 in the IFN-γ producing CD8 T cells specific for TB10.4 (p<0.001) (Fig 2A and C). Moreover, when summing up the T cells expressing all combinations of IFN-γ, TNF-α, and IL-2 we found that in the rH4/Ad-H4 group 6.9% of all CD8 T cells were specific for Ag85B and 19.6% for TB10.4. Both vaccines induced IFN-γ^+^TNF-α^+^ double positive CD8 T cells specific for both Ag85B and TB10.4, and interestingly also a minor, but still detectable, population of triple positive antigen specific CD8 T cells (Fig. 2B and D). Consistent with this, stimulation of splenocytes from the vaccinated mice *in vitro* for 72 hours (instead of 6 hours in the FACS analysis) followed by ELISA analysis of IFN-γ secretion, also revealed significantly higher IFN-γ production in the rH4/Ad-H4 vaccine group, although mice from all vaccine groups gave measurable responses (Fig. 2E). Taken together, we found that heterologous prime boost based on rH4 and Ad-H4 had a clear additive effect on the number of vaccine antigen specific CD4 and CD8 T cells, inducing higher numbers of polyfunctional CD4 and CD8 T cells compared to rH4 and Ad-H4 vaccinations.

### Vaccine induced cytotoxicity

**Figure 3:**
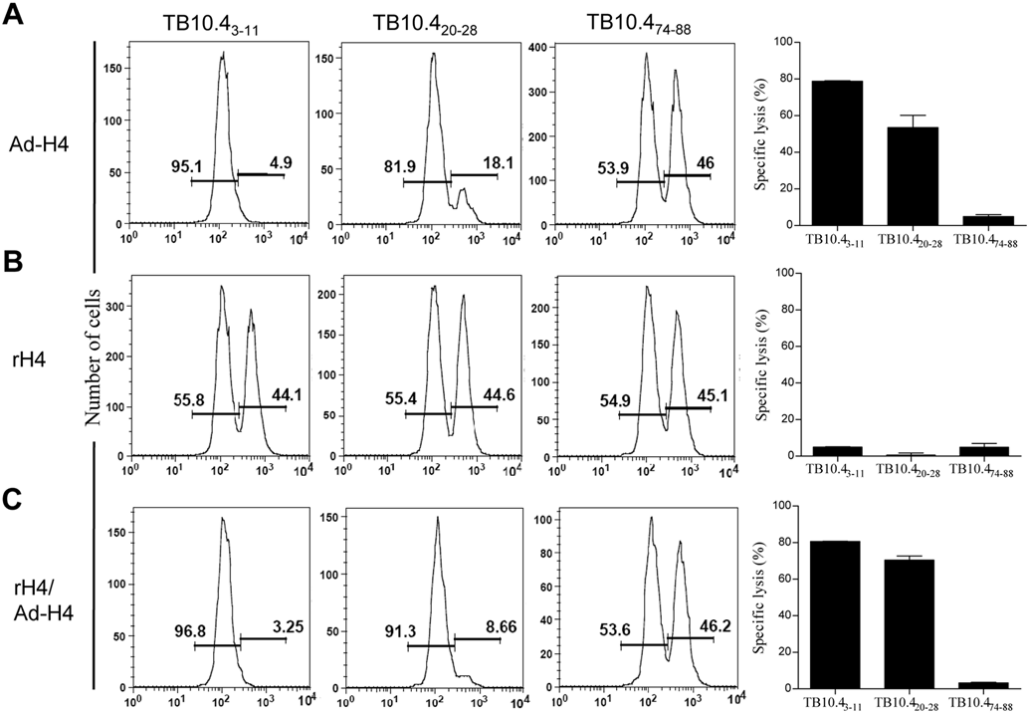
Functional characterization of the vaccine induced T cells. The specific lysis of TB10.4_3-11_, TB10.4_20-28_, and TB10.4_70-88_ loaded cells were determined in an *in vivo* cytotoxicity assay. Unloaded (CFSE^low^) and TB10.4_3-11_, TB10.4_20-28_, or TB10.4_70-88_ (CFSE^high^) loaded splenocytes from naïve mice were transferred into Ad-H4 (A), rH4 (B), or rH4/Ad-H4 (C) vaccinated mice. (A–C) The amount of splenocytes killed *in vivo* by cytotoxic T cells specific for either of the TB10.4 target peptides was observed as a reduction in the CFSE^high^ population Percent specific lysis was calculated and is shown in the far right graphs. Non-specific cytotoxicity from non-vaccinated mice has been subtracted (Non-specific killing in non-vaccinated mice of either CFSEhigh or CFSElow cells were from 5–15%).

To further characterize the vaccine induced T cells we next analyzed the cytotoxic potential of the induced CD8 (and CD4) T cells. We used the recently published MHC class I restricted epitopes (specific for TB10.4_3-11_ and TB10.4_20-28_) and one MHC class II restricted epitope (TB10.4_70-88_) [11], [21], [26]. CFSE labeled splenocytes from naïve mice, pulsed (CFSE high) or not pulsed (CFSE low) with either of the two MHC class I restricted peptides or the MHC class II restricted epitope from TB10.4, were adoptively transferred into mice that had been vaccinated with rH4/Ad-H4 a week before. For comparison we included Ad-H4 and rH4 vaccinated mice. After 4 hours peptide specific lysis of the transferred cells was determined by FACS analysis of recipient splenocytes. We observed 78.8%±0.2 and 80.6%±0.2 specific killing of the TB10.4_3-11_ loaded target cells (CFSE high) in Ad-H4 and rH4/Ad-H4 mice, respectively, compared to non-loaded target cells (CFSE low) (p<0.05). The rH4/Ad-H4 mice also demonstrated strong cytotoxic potential against TB10.4_20-28_ loaded target cells with 70.5%±2.3 specific killing, which was slightly higher than the TB10.4_20-28_ specific killing in Ad-H4 mice (53.5%±6.6, p<0.05) (Fig. 3A and C). In contrast, there was no killing of target cells loaded with the MHC II restricted epitope TB10.4_70-88_, and the rH4 group did not demonstrate any cytotoxic killing of the transferred target cells (Fig. 3B). Thus, both Ad-H4 and rH4/Ad-H4 induced CD8 T cells with a cytotoxic potential.

### The protective efficacy of the vaccine induced T cell responses

**Figure 4:**
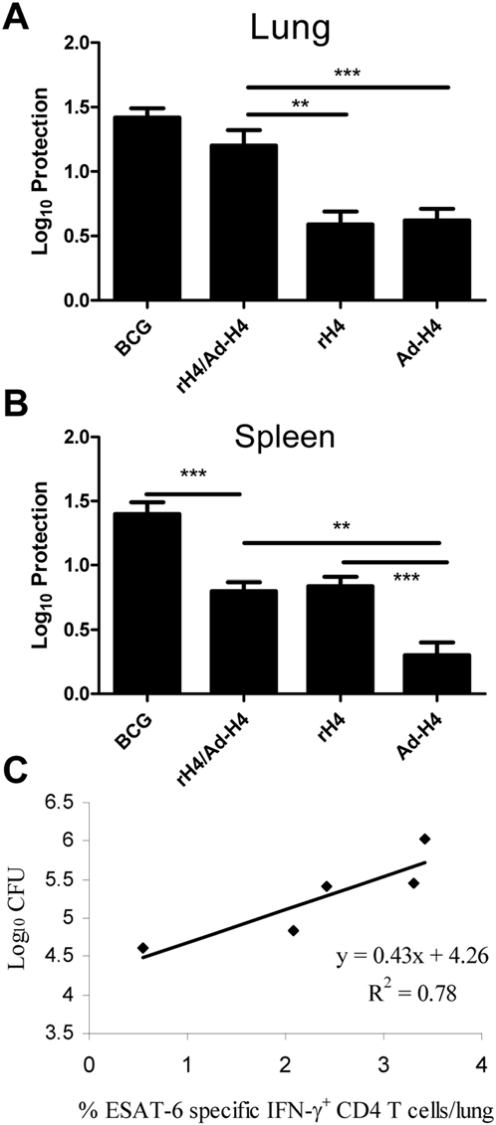
The protective efficacy and correlation of protection with infection driven immune response in the vaccinated and challenged mice. (A and B) Protection in vaccinated mice (expressed as Log_10_ CFU reduction) compared to unvaccinated and BCG vaccinated controls challenged by the aerosol route with virulent *M.tb.* six weeks after the final vaccination. Six weeks post-challenge, the mice were sacrificed and the bacterial burden (CFU) was measured in the lung or spleen. Data represent the mean+SEM of a minimum of five mice per group in two individual experiments with ***, p<0.001 and **, p<0.01 (2-way ANOVA with Bonferroni posttest). (C) Relation between protection (CFU) and ESAT-6 specific IFN-γ producing CD4 T cells is shown as a fitted regression line with the correlation coefficient (R^2^). Six weeks post challenge, the mice were sacrificed and the lung cells were stimulated with ESAT-6 overlapping peptides prior to staining with anti-CD4, -CD44, and –IFN-γ. Frequencies represent IFN-γ producing ESAT-6 specific CD44^+^CD4^+^ T cells out of total CD4 T cells. Background staining from cells stimulated with medium alone has been subtracted. Data represent the mean of +SEM of a minimum of five mice per group in two individual experiments.

We next analyzed the vaccine induced protection. Vaccinated CB6F1 mice were challenged six weeks after final vaccination, and six weeks post challenge, the mice were sacrificed and the bacterial numbers were determined in the lungs. Ad-H4, rH4, the hetrologous prime boost combination, non-vaccinated, and BCG vaccinated mice were included. All the H4 based vaccines induced significant protection against *M.tb* challenge but as observed with the T cell response, combining the two vaccines, rH4 and Ad-H4, showed an additive effect in terms of protection. Thus, mice vaccinated with the heterologous prime boost combination of rH4/Ad-H4 showed a bacterial reduction of 1.2 Log_10_±0.1 CFU, which was significantly higher than the protection in Ad-H4 and rH4 vaccinated mice (p<0.01 and p<0.001, respectively). The BCG-vaccinated mice showed a protection of 1.42 Log_10_±0.1 CFU, which was not significantly different from rH4/Ad-H4 vaccinated mice (Fig. 4A). Interestingly, the CFU levels at six weeks post infection in the vaccine groups directly correlated with the ESAT-6 CD4 T cell response (R^2^ = 0.78) (Fig. 4B), in agreement with previous work indicating that the ESAT-6 specific response can be used as a predictor for bacterial load [23]. In the spleen, the highest protection was seen in BCG vaccinated mice, and in the groups vaccinated with either rH4 or rH4/Ad-H4.

### Recruitment of vaccine induced antigen specific CD4 and CD8 T cells to the site of infection

**Figure 5:**
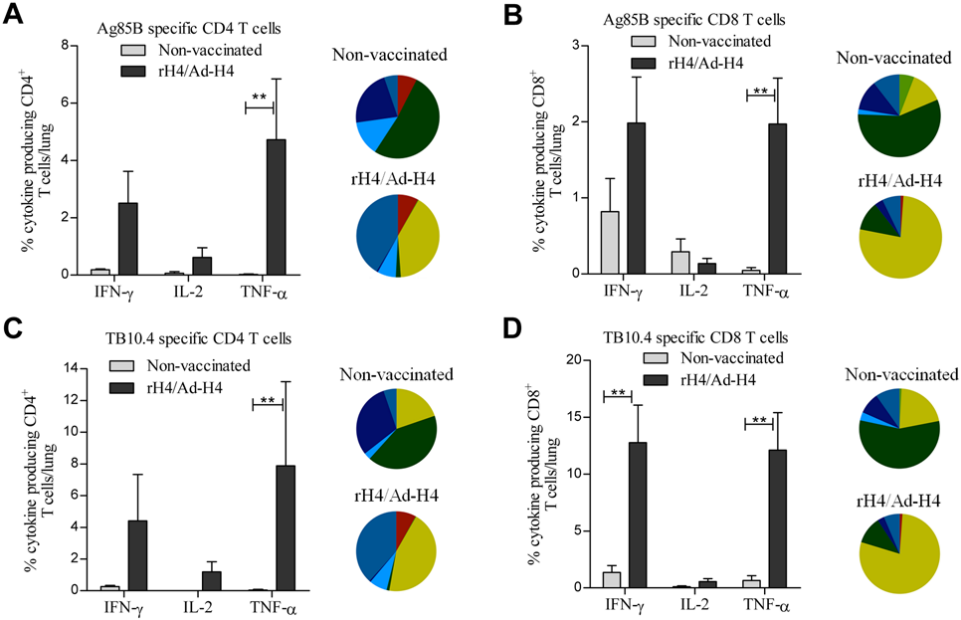
Cytokine frequencies and phenotypic profiles of specific CD4 and CD8 T cells in non-vaccinated or rH4/Ad-H4 vaccinated mice two weeks after challenge. CB6F1 mice were vaccinated once with rH4 in cationic liposomes followed two weeks later by one Ad-H4 vaccination, and challenged by the aerosol route with virulent *M.tb* six weeks after the final vaccination. Two weeks after challenge the mice were sacrificed and lung lymphocytes were stimulated with Ag85B or TB10.4 peptides prior to staining with anti-CD4, -CD8, -CD44, -IFN-γ, -IL-2, and –TNF-α. Frequencies represent IFN-γ, IL-2, or TNF-α producing CD44^+^CD4^+^ or CD44^+^CD8^+^ T cells specific for Ag85B (A and B) or TB10.4 (C and D). Background staining from cells stimulated with medium alone has been subtracted. Data represent the mean+SEM of four mice per group with **, p<0.01 (2-way ANOVA with Bonferroni posttest). IFN-γ, IL-2, and TNF-α cytokine profiles of Ag85B or TB10.4 specific CD4 and CD8 T cells are shown in pies as triple, double, or single positive CD4 or CD8 T cells. The color code for the pies is the same as shown in Fig. 1E.

As we had observed an increase in both CD4 and CD8 T cells in the group receiving the heterologous prime boost combination of rH4/Ad-H4, and increased protection, it was important to compare how the vaccine primed CD4/CD8 T cells responded to an *M.tb* infection (in the group where they were both induced, e.g. the rH4/Ad-H4 group). As controls, non-vaccinated mice were included. Mice vaccinated with rH4/Ad-H4 (or non-vaccinated) were challenged with virulent *M.tb* six weeks after last vaccination, and two weeks after infection the mice were sacrificed. The two weeks post infection time point was chosen since the majority of cells in the lung at that time point are recalled T cells and not yet newly infection-primed T cells (Non vaccinated showed <1% CD8 Ag85B or TB10.4 specific T cells and <0.2% CD4 T cells) [23], which enabled us to measure recruitment of the pre-existing CD4 and CD8 T cells. Lung lymphocytes were stimulated *in vitro* with Ag85B and TB10.4, and expression of IFN-γ, IL-2, and TNF-α in the CD4 and CD8 T cell subsets was analyzed by intracellular FACS. Two weeks post infection we observed a significant higher proportion of Ag85B and in particular of TB10.4 specific CD4 T cells in the lungs in the rH4/Ad-H4 group when compared to the non-vaccinated group (p<0.01) (Fig. 5A and C). There were also increased levels of CD8 T cells in the lungs of rH4/Ad-H4 vaccinated mice, in particular of the IFN-γ^+^ TNF-α^+^ effector phenotype (Fig. 5B and D) [24], [25]. Of all CD8 T cells 13.8% were specific for TB10.4 and 2.4% for Ag85B in the vaccinated group, and the numbers for CD4 T cells were 8.0% and 4.8%, respectively (Table 1). Thus, both CD4 and CD8 T cells were recruited to the lungs. Importantly, the CD8 T cells induced in the non-vaccinated mice showed a much higher frequency of single positive T cells, as compared to the vaccinated group, indicating the induction of terminally differentiated T cells by the infection alone (Fig. 5B and D). Interestingly, in the rH4/Ad-H4 group more than 50% of all the cytokine producing CD4 T cells had a more memory-like phenotype (IFN-γ^+^IL-2^+^TNF-α^+^, IL-2^+^TNF-α^+^ or IL-2^+^) [24], [25], in comparison with only 10–20% of all the CD8 T cells (Table 1).

**Table 1:** Frequency of T cell subsets.

T cells (%)***	Two weeks pre-infection*				Two weeks post Infection**			
	Ag85B		TB10.4		Ag85B		TB10.4	
	CD4	CD8	CD4	CD8	CD4	CD8	CD4	CD8
IFN-γ^+^ IL-2^+^ TNF-α^+^	0.44	0.06	0.01	0.04	0.40	0.04	0.67	0.18
IFN-γ^+^ IL-2^+^ TNF-α^−^	0.00	0.01	0.00	0.02	0.01	0.00	0.03	0.00
IFN-γ^+^ IL-2^−^ TNF-α^+^	0.34	1.13	0.03	3.29	1.99	1.68	3.56	10.99
IFN-γ^+^ IL-2^−^ TNF-α^−^	0.03	0.81	0.00	2.11	0.11	0.27	0.10	1.46
IFN-γ^−^ IL-2^+^ TNF-α^+^	0.37	0.03	0.01	0.96	0.30	0.00	0.56	0.02
IFN-γ^−^ IL-2^+^ TNF-α^−^	0.00	0.00	0.00	0.00	0.00	0.11	0.00	0.32
IFN-γ^−^ IL-2^−^ TNF-α^+^	0.51	0.66	0.10	0.64	2.03	0.25	3.10	0.89
In total	1.69	2.69	0.15	7.05	4.84	2.35	8.03	13.85
	Two weeks pre-infection*				Two weeks post Infection**			
CD4 T cell (%)***		15.1				25.1		
CD8 T cell (%)		18.0				19.4		

Frequency of cytokine producing Ag85B and TB10.4 specific CD4 and CD8 T cells two weeks before and after *M.tb* challenge, as well as the frequencies of total CD4 and CD8 T cells, in rH4/Ad-H4 vaccinated mice.

* The frequencies were obtained by FACS analysis on *in vitro* antigen stimulated splenocyte cultures from vaccinated mice (two weeks before challenge).

** The frequencies were obtained by FACS analysis on *in vitro* antigen stimulated lung lymphocyte cultures from vaccinated and *M.tb* challenged mice two weeks after challenge.

*** Background staining from cells stimulated with medium alone has been subtracted.

**Figure 6:**
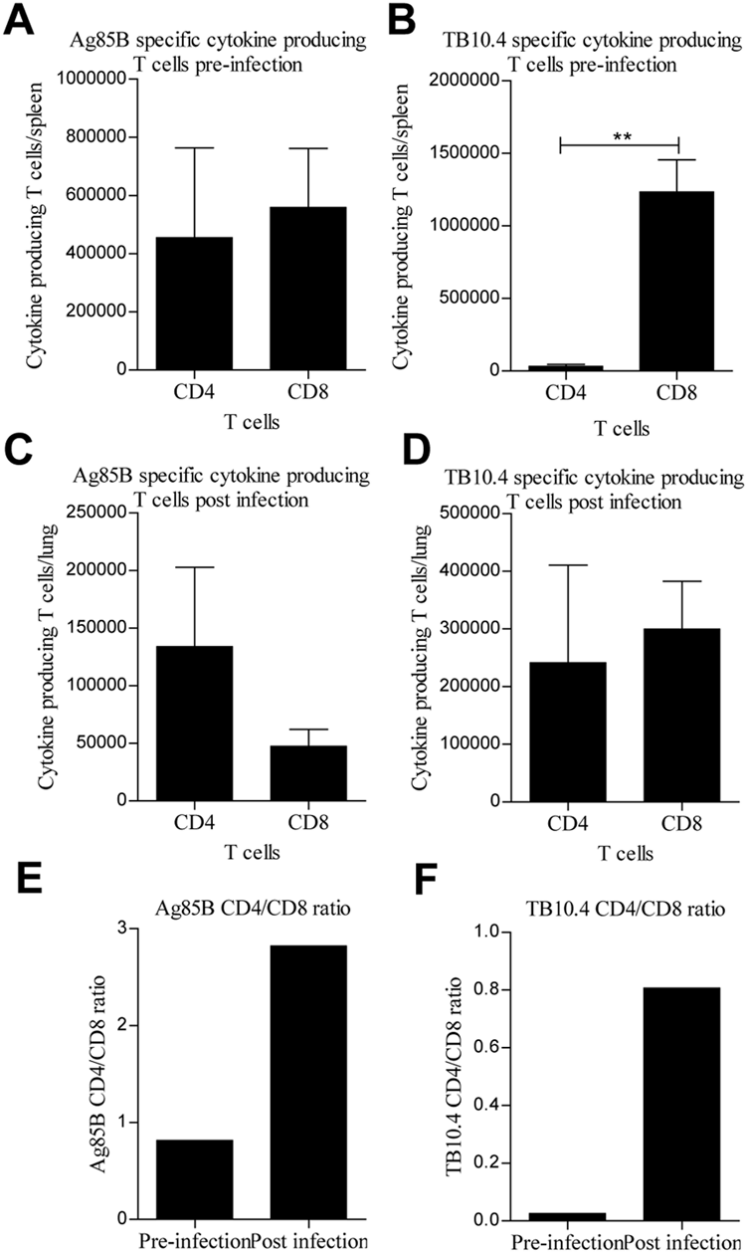
Recruitment of Ag85B and TB10.4 CD4 and CD8 T cells to the lung. Antigen specific CD4 and CD8 T cells in rH4/Ad-H4 vaccinated mice were measured two weeks before and after challenge. CB6F1 mice were vaccinated once with rH4 in cationic liposomes followed two weeks later by one Ad-H4 vaccination, and challenged by the aerosol route with virulent *M.tb* six weeks after the final vaccination. Two weeks before challenge and two weeks after challenge mice were sacrificed, and splenocytes and lung lymphocytes, respectively, were stimulated with Ag85B or TB10.4 peptides prior to staining with anti-CD4, -CD8, -CD44, -IFN-γ, -IL-2, and –TNF-α. (A–D) The number of cytokine producing T cells is a total number of triple, double, and single positive IFN-γ, TNF-α, and IL-2 producing antigen specific CD4 or CD8 T cells either per spleen pre-infection (A and B) or per lung post infection (C and D). Background staining from cells stimulated with medium alone has been subtracted. Data represent the mean+SEM of four mice per group with **, p<0.01 (2-way ANOVA with Bonferroni posttest). (E and F) The ratio between Ag85B (E) and TB10.4 (F) specific CD4 and CD8 T cells was calculated from A–D.

To analyze whether there was a more extensive recruitment, or expansion, of CD4 versus CD8 T cells, we next quantified the cell proportions of Ag85B and TB10.4 specific CD4 or CD8 T cells before and after infection. To determine the numbers of the antigen specific cells, all the subpopulations were added to give the total number of T cells expressing any combination of IFN-γ, TNF-α, and IL-2 (Table 1). Pre-infection (two weeks pre-infection) we observed equal amounts of Ag85B specific CD4 and CD8 T cells in the vaccinated group in the spleen (Fig. 6A). However, 2 weeks post infection there were 2½ times more Ag85B CD4 T cells than CD8 T cells in the lungs (Fig. 6C and E). As for TB10.4 specific T cells, around 50 times more TB10.4 specific CD8 T cells were observed pre-infection in the spleen than CD4 T cells (TB10.4 CD4/CD8 ratio of 0.025) (Fig. 6B). However, after infection we found almost the same numbers of TB10.4 CD4 and CD8 T cells in the lungs (CD4/CD8 ratio of 0.80) (Fig. 6D and F). This demonstrated that the CD4 T cells in both the Ag85B and TB10.4 pool of T cells were initially recruited/expanded more than the CD8 T cells, even though both cell types were clearly recruited to the lung at the onset of the infection.

### The proliferative capacity of the recruited T cells at the site of infection

**Figure 7:**
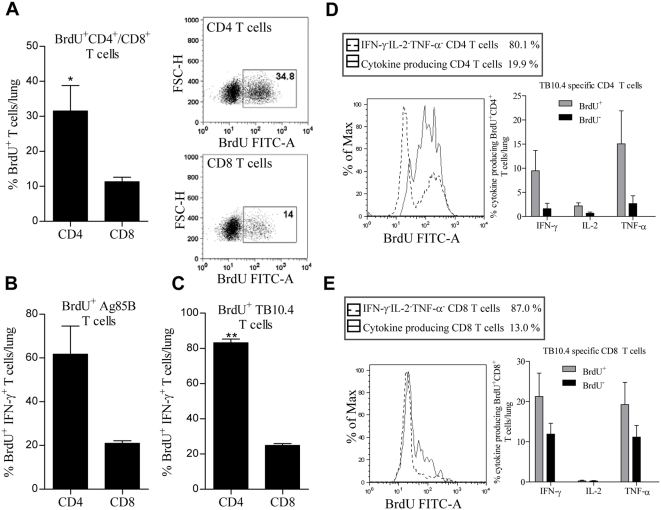
The proliferative capacity and frequency of the recruited Ag85B and TB10.4 specific CD4 and CD8 T cells to the lungs. CB6F1 mice were vaccinated once with rH4 in cationic liposomes followed two weeks later by one Ad-H4 vaccination, and challenged by the aerosol route with virulent *M.tb* at week 10. The last three days before killing, BrdU (0.8 mg/ml) was added to the drinking water. Two weeks after challenge, mice were sacrificed and lung lymphocytes were stimulated with Ag85B or TB10.4 peptides prior to staining with anti-CD4, -CD8, -BrdU, and -IFNγ (A–C). (A) Frequencies represent the percentage of BrdU^+^ CD4 or CD8 T cells out of total CD4 or CD8 T cells. Data represent the mean of +SEM of four mice per group with *, p<0.05 (Unpaired t-test, two-tailed). Dot plots, representative of four mice, are also shown depicting BrdU^+^ CD4 or CD8 T cells. (B and C) Frequencies represent the percentage of BrdU^+^ CD4 or CD8 T cells out of total IFN-γ^+^ CD4 or CD8 T cells specific for either Ag85B (B) or TB10.4 (C). Data represent the mean+SEM of two mice per group with **, p<0.01 (Unpaired t-test, two-tailed). Background staining from cells stimulated with medium alone has been subtracted. (D and E) CB6F1 mice were vaccinated once with rH4 in cationic liposomes followed two weeks later by one Ad-H4 vaccination, and challenged by the aerosol route with virulent *M.tb* at week 10. The last three days before sacrificing the animals, BrdU (0.8 mg/ml) was added to the drinking water. Two weeks after challenge mice were sacrificed and lung lymphocytes were stimulated with TB10.4 peptides prior to staining with anti-CD4, -CD8, -BrdU, -IFN-γ, TNF-α, and IL-2. The BrdU histograms represent a normalized overlay of all the cytokine producing CD4 (D) and CD8 (E) T cells, (IFN-γ^+^IL-2^+^TNF-α^+^, IFN-γ^+^TNF-α^+^, IFN-γ^+^IL-2^+^, IFN-γ^+^, IL-2^+^TNF-α^+^, IL-2^+^, and TNF-α^+^) (solid line), and all the non-cytokine producing CD4 and CD8 T cells (IFN-γ^−^IL-2^−^TNF-α^−^) (dashed line), and the percentages shown are the percentages of cytokine producing or cytokine non-producing T cells out of total T cells. Instead of displaying counts in the FACS histogram overlays, each histogram in the overlay has been normalized to its maximum counts (“% of MAX” counts). Data represent the mean of +SEM of three mice per group. Background staining from cells stimulated with medium alone has been subtracted.

Since CD4 T cells were preferentially recruited to the infection site, we next evaluated if these infiltrating CD4 T cells were the result of an increased turn-over rate of CD4 versus CD8 T cells. The vaccinated and challenged mice were given BrdU in the drinking water three days before sacrifice to measure the degree of proliferation of CD4 and CD8 T cells at two weeks post infection. Of all CD4 T cells 31.5%±7.3 had incorporated BrdU, whereas this number was 11.3%±1.3 (p<0.05) for CD8 T cells (Fig. 7A). Stimulation with TB10.4 or Ag85B followed by gating on the IFN-γ positive antigen specific T cells showed that also for antigen specific T cells the CD4 T cell population showed the highest proliferation (Fig. 7B and C). Gating on all combinations of T cell subtypes expressing one or more of the cytokines IFN-γ/IL-2/TNF-α clearly demonstrated that the majority of proliferating CD4 cells were cytokine expressing, in contrast to the non-cytokine producing CD4 T cell population, which did not proliferate (IFN-γ^−^IL-2^−^TNF-α^−^, Fig. 7D). In contrast, for the CD8 T cell population, the majority of the cytokine producing cells were non-proliferating (Fig. 7E). Moreover, when looking at the frequency of cytokine producing CD4 T cells, we found a clear tendency towards higher IFN-γ, IL-2, and TNF-α production by the BrdU^+^ CD4 T cells compared to the BrdU^−^ CD4 T cell population (Fig. 7D). This tendency was less pronounced, however still present, in the cytokine producing antigen specific CD8 T cells (Fig. 7E). In conclusion, both the vaccine induced CD4 and CD8 T cells undergo cell division and were recruited to the site of infection, at the onset of the infection. However, the CD4 T cells showed a higher frequency of memory-like phenotypes, were recruited to the lungs at a higher rate, and proliferated more than CD8 T cells.

## Discussion

CD4 T cells are crucial for defense against *M.tb.* However, it is still not clear whether the optimal response against infection with *M.tb* in fact involves both CD4 and CD8 T cells and if these subsets play a role in distinct and different stages of the infection [27]. In the present study we therefore compared the well characterized vaccine fusion molecule rH4 (Ag85B-TB10.4) delivered either as a subunit vaccine in cationic liposomes (rH4/CAF01) expressed in an adeno5 vector (ad-H4) or as a heterologous prime boost combination. We clearly show that rH4/CAF01 induces primarily CD4 T cells, and Ad-H4 CD8 T cells, but the heterologous prime boost combination induces both CD4 and CD8 T cells.

Although CD8 T cells are a significant part of the immune response induced by the natural infection, the role of CD8 T cells in the defense against *M.tb* is not fully resolved. Lately, several vaccine strategies have made use of viral vectors to deliver the vaccine antigens, and to induce vaccine specific CD8 T cells [28]–[30]. These studies all point to a protective role for CD8 T cells, even though in most of these studies CD4 T cells were also primed. Studies using MVA as delivery of the vaccine antigens have indicated that this vector is particularly efficient when used as a booster vaccine, and boosting of both CD4 and CD8 T cells have been observed [15], [31]. Likewise, adenoviral vectors have been found to serve as efficient boosters of existing immune responses. Thus, adenovirus expressing Ag85A was able to boost BCG immunity, which in turn increased the protection against *M.tb* [32]. As with MVA, adenovirus boosted both the CD4 and CD8 T cell activity, and priming of both these subsets was also observed when following immunization with adenovirus expressing mycobacterial antigens [18], [29], [33]. In other recent studies, in which only CD8 T cells to immunodominant antigens (such as ESAT-6 or TB10.4) were induced, only minor or no protection was observed [17], [21], indicating that the contribution of CD8 T cells to protection may depend on, or increase inthe presence of, an ongoing CD4 T cell response. This is in line with studies showing that both CD8 T cell memory induction and maintenance can be influenced by CD4 T cells [34]–[40]. However, it should be mentioned that in two studies using vaccination with a DNA vaccine encoding CFP10, or a mix of several TB proteins, the vaccine induced CD8 T cells were shown to induce protection, demonstrating that in some instances CD8 T cells do provide protection against acute *M.tb* infection in a manner that is not strictly dependent upon simultaneous induction of CD4 T cells [5], [41].

In the present study we wanted to study the potential of a heterologous prime boost vaccine strategy that induce both CD4 and CD8 T cells, and to compare the phenotype of vaccine primed CD4 and CD8 T cells, as well as the recruitment and proliferation of these cells following infection with *M.tb.* We combined vaccination of a CD4 T cell inducer, rH4/CAF01, with a strong CD8 T cell inducer, Ad-H4. Mice vaccinated with the rH4/Ad-H4 combination showed an increase in CD4 T cell activity indicating that rH4 primed CD4 T cells can be boosted by Ad-H4 immunization. Compared to Ad-H4, rH4/Ad-H4 also induced significant higher numbers of cytokine producing CD8 T cells. In the rH4/Ad-H4 group approximately 27% of all CD8 T cells were specific for the vaccine antigens, with 7% being specific for Ag85B and 20% for TB10.4. Most of these (45–80%) CD8 T cells expressed IFN-γ and TNF-α (Fig. 1 and 2) which suggests an effector phenotype. This clearly demonstrated that combining rH4 and Ad-H4 had a strong additive effect on priming for both the CD4 and CD8 T cell response. It can be speculated that the boosting of the CD4 T cells, which occurred in parallel with the priming of CD8 T cells, provided increased CD4 help for the CD8 T cells, thereby further promoting the activation and expansion of these cells. We also observed a slightly increased cytolytic function of the CD8 T cells in the rH4/Ad-H4 group, in particular against TB10.4 _20-28_ loaded target cells (17% increase in the specific killing of these cells in the rH4/Ad-H4 group cpmpared to the Ad-H4 group) (Fig. 3). This may be due to increased CD8 T cell numbers, increased cytolytic capacity of the individual T cells, or that the CD8 T cells in the rH4/Ad-H4 group were primed in the presence of CD4 T cells, which have been suggested to increase both the persistence of CD8 T cells and the ability of the CD8 cells to respond to a secondary encounter with their antigen [34]–[38].

In terms of protection against infection with *M.tb*, we found, as observed with the numbers of vaccine specific T cells (Fig. 1 and 2), that combining the two vaccine strategies also had a significant additive effect in the lung. Thus, mice vaccinated with rH4/Ad-H4 showed a protection of 1.2 Log_10_±0.1 CFU, which was significantly higher than the protection observed in the Ad-H4 and rH4 mice (Fig. 4A) and not significantly different from that observed in BCG vaccinated mice. The Ad-H4 group showed a protection of approximately 0.4 Log_10_ CFU, which is in agreement with a recent study using Ad-35-Ag85A-Ag85B-TB10.4 [18]. Interestingly, in the spleen, the lowest protection was observed in the Ad-H4 group, which may indicate that prevention of dissemination from the lung to the spleen require CD4 T cells more than CD8 T cells (Fig. 4B).

In the rH4/Ad-H4 group two weeks after infection we observed that 14% of all CD8 T cells in the lungs were specific for TB10.4 and 2–3% for Ag85B, indicating that the CD8 epitopes were indeed processed and presented in the lungs at the very onset of infection, and that CD8 T cells primed by Ad5 vector delivery, as well as the CD4 T cells, were recruited to the site of infection (pre-infection the numbers in the lung were less than 0.1%, data not shown) (Fig. 5). For both the CD4 and CD8 T cell subsets we observed more polyfunctional cells (IFN-γ^+^TNF-α^+^ or IFN-γ^+^TNF-α^+^IL-2^+^) in the rH4/Ad-H4 group following *M.tb* challenge compared non-vaccinated mice. This could indicate that this vaccination strategy resulted in a pool of polyfunctional T cells that was maintained after challenge, whereas the natural infection of unprimed mice tended to drive the activated T cells toward a state of terminal differentiation (IFN-γ^+^) (Fig. 5) [25]. Interestingly, in the rH4/Ad-H4 vaccinated mice, we noted important differences between the CD4 and CD8 T cell recall responses. Thus, although we observed strong, local CD8 T cell memory, as measured by total CD8 T cell numbers, CD4 T cells preferentially accumulated (due to recruitment and/or expansion) at the site of infection (Fig. 6). In line with this, CD4 T cells with proliferative capacity showed higher accumulation at the site of infection than CD8 T cells two weeks post infection (although we can exclude that this bias in CD4 proliferation may in part reflect a bias inherent in the CD4 prime/boost mechanism). This was observed by analyzing the total pool of CD4 T cells, and also when studying TB10.4 and Ag85B antigen specific T cells (Fig. 7). Moreover, as for the phenotype of the proliferating cells, the majority of the cytokine (any combination of IFN-γ, TNF-α or IL-2) producing CD4 T cells belonged to the proliferating CD4 T cell population, in contrast to the cytokine producing CD8 T cells that where mainly non-proliferating (Fig. 7). Taken together, these data revealed that although both CD4 and CD8 T cells were primed through vaccination, there was an increased recruitment and proliferation of the CD4 T cells as compared to the CD8 T cells suggesting that this subset may be primarily involved in the recall response that results in early control of bacterial replication in vaccinated mice, and that increasing the CD4 T cell numbers in this stage of the infection is important. We are presently testing H4 in new adjuvants specifically selected for their ability to induce a strong CD4 T cell response.

In conclusion, heterologous prime boost based on H4, produced an additive effect on the priming of CD4 and CD8 cells and in terms of the protective capacity of the vaccine, and therefore represent an interesting vaccine strategy against *M.tb*. However, compared to CD8 T cells, antigen specific CD4 cells were more polyfunctional, recruited at a higher rate, and proliferated more following infection with *M.tb*, indicating that although both CD4 and CD8 epitopes are clearly presented at the very onset of infection, CD4 and CD8 T cells respond very differently to live infection, in a manner which support the consensus that CD4 T cells play the major role during the early stages of an *M.tb* infection.

## Materials and Methods

### Ethics Statement

The handling of mice were conducted in accordance with the regulations set forward by the Danish Ministry of Justice and animal protection committees by Danish Animal Experiments Inspectorate, and in compliance with European Community Directive 86/609 and the U.S. Association for Laboratory Animal Care recommendations for the care and use of laboratory animals.

### Animals

Studies were performed with six-eight week-old female CB6F1 (C57BL/6×BALB/c) mice from Harland Netherlands. Non-infected mice were housed in cages in appropriate animal facilities at Statens Serum Institut or at the Panum Institute, University of Copenhagen. Infected animals were housed in cages contained within laminar flow safety enclosures (Scantainer from Scanbur, Denmark) in a separate biosafety level 3 facility at Statens Serum Institut. All mice were fed radiation sterilized 2016 Global Rodent Maintenance diet (Harlan, Scandinavia) and water *ad libitum*. All animals were allowed a one-week rest before initiation of the experiments.

### BrdU

In experiments measuring proliferation, BrdU (0.8 mg/ml) was added to the drinking water to the mice for three days before sacrifice.

### Bacteria

*M.tb* Erdman was grown at 37°C in suspension in Sauton medium (BD Pharmingen) enriched with 0.5% sodium pyruvate, 0.5% glucose, and 0.2% Tween 80. All bacteria were stored at −80°C in growth medium at ∼5×10^8^ CFU/ml. Bacteria were thawed, sonicated, washed, and diluted in phosphate-buffered saline (PBS) for immunizations and infections. All bacterial work was performed at the Statens Serum Institut by authorized personnel.

### Construction of recombinant replication-deficient adenoviral 5 based vaccine Ad-H4

The *M.tb* fusion protein H4 consisting of the two antigens Ag85B and TB10.4 was amplified by overlapping PCR from plasmid template and cloned into a pacCMV-based shuttle vector. From the shuttle plasmid, human type 5 recombinant non-replicating adenovirus vectors were produced from homologous recombination by standard methods [42]. After purification, adenoviral stocks were immediately aliquoted and frozen at −80°C in 10% glycerol, and the infectivity of the adenoviral stocks was determined with Adeno-X Rapid Titer kit (BD Clontech).

### Immunization

Mice immunized with Ad-H4 were anesthetized and injected once with 2×10^7^ infectious units (IFU) s.c. in the right hind footpad. Mice immunized with the Ag85B-TB10.4 fusion protein (rH4) were immunized twice at two-week intervals s.c. at the base of the tail with a total volume of 200 µl containing 5 µg rH4 formulated in cationic liposomes (CAF01). For boosting studies, mice received a single dose of rH4 injected s.c. at the base of the tail and two weeks later the mice were given one booster vaccination of Ad-H4 in the right footpad. Non-vaccinated control mice received NaCl s.c. at the base of the tail, and the BCG control mice received a single dose of BCG Danish 1331 (2.5×10^5^ CFU) injected s.c. at the base of the tail.

### Experimental infections

When challenged by the aerosol route, the animals were infected with ∼50 CFU of *M.tb* Erdman/mouse with an inhalation exposure system (Glas-Col, Indiana, USA). Mice were challenged eight weeks after the first vaccination and killed either two weeks post-challenge for immune surveillance or six weeks post-challenge for bacterial counts. Numbers of bacteria in the lung were determined by serial three-fold dilutions of individual whole-organ homogenates in duplicate on 7H11 medium. Colonies were counted after two-three weeks of incubation at 37°C. Protective efficacies were expressed as log_10_ bacterial CFU.

### Lymphocyte preparation

Splenocytes and lung lymphocytes were obtained by passage of the organs through a 100 µm nylon cell strainer (BD Pharmingen, USA) followed by two washing procedures using RPMI 1640. Cells in each experiment were cultured in sterile microtiter wells (96-well plates; Nunc, Denmark) containing ∼2×10^6^ cells in 200 µl of RPMI 1640 supplemented with 1% (v/v) premixed penicillin-streptomycin solution (Invitrogen Life Technologies), 1 mM glutamine, and 5% (v/v) fetal calve serum (FCS) at 37°C/5% CO_2_. The lymphocyte cultures were further used for ELISA and FACS analysis.

### Antigens for in vitro stimulation

Synthetic overlapping peptides (9- and 18-mers) covering the complete primary structure of Ag85B, TB10.4, and ESAT-6 were synthesized by standard solid-phase methods on a SyRo peptide synthesizer (MultiSynTech, New England) at the JPT Peptide Technologies (Berlin, Germany), or at Schafer-N (Copenhagen, Denmark). Peptides were lyophilized and stored dry at −20°C until reconstitution in PBS.

### IFN-γ enzyme-linked immunosorbent assay (ELISA)

Microtiter plates (96 well; Maxisorb; Nunc, Denmark) were coated with 1 µg/ml monoclonal rat anti-murine IFN-γ (clone R4-6A2; BD Pharmingen). Free binding sites were blocked with 2% (w/v) milk powder in PBS. Culture supernatants were harvested from lymphocyte cultures after 72 hours of *in vitro* antigen stimulation and tested in triplicates. IFN-γ was detected with a 0.1 µg/ml biotin-labeled rat anti-murine antibody (mAb; clone XMG1.2; BD Pharmingen, USA) and 0.35 µg/ml horseradish peroxidase-conjugated streptavidin (Zymed, Invitrogen, USA). The enzyme reaction was developed with 3.3′, 5.5′-tetramethylbenzidine, hydrogen peroxide (TMB *plus;* Kementec, Denmark) and stopped with 0.2 M H_2_SO_4._ rIFN-γ (BD Pharmingen, USA) was used as a standard. Plates were read at 450 nm with an ELISA-reader and analyzed with KC4 3.03 Rev 4 software.

### Flow cytometric analysis

Intracellular cytokine staining procedure: Splenocytes or lung lymphocytes were stimulated for 1 h with 2 µg/ml antigen at 37°C/5% CO_2_ and subsequently incubated for 5 h at 37°C with 10 µg/ml brefeldin A (Sigma-Aldrich, USA) at 37°C. The cells were washed in FACS buffer (PBS containing 0.1% sodium azide and 1% FCS) before surface staining with rat anti-mouse antibodies. Cells were washed with FACS buffer before fixation and permeabilization using the BD Cytofix/Cytoperm™ (BD, San Diego, CA, USA) according to the manufacturer's protocol followed by intracellular staining. When staining with FITC-anti-BrdU an extra fixation and permabilisation step, as well as DNAse treatment, was introduced before the intracellular staining. Cells were washed and resuspended in formaldehyde solution 4% (w/v) pH 7.0 (Bie & Berntsen, Denmark) and samples were analyzed on a six-color BD FACSCanto flow cytometer (BD Biosciences, USA). Data analysis was done on FlowJo Software (© Tree Star, Asland, OR, USA). The following antibodies were used for surface staining: PerCP-Cy.5.5-anti-CD8α (53-6.7), APC-Cy7-anti-CD4 (GK1.5), and FITC-anti-CD44. For intracellular staining the following antibodies were used: PE-anti-TNF-α, APC-anti-IL-2, PE-Cy7-anti-IFN-γ, and FITC-anti-BrdU. All antibodies were purchased from BD Pharmingen (San Diego, USA) or eBiosciences (San Diego, USA).

### In vivo cytotoxicity assessed by adoptive transfer of CFSE-labeled target cells

Single splenocyte suspensions of CB6F1 mice were obtained by passage through a fine 100 µm nylon cell strainer (BD Pharmingen, USA). Erythrocytes were depleted by lysis in ammonium chloride solution, washed in PBS before resuspension in incomplete RPMI and stained with 5(6)-Carboxyfluorescein diacetate *N*-succinimidyl ester (CFSE) (Sigma-Aldrich, San Louis, USA) at CFSE^high^ (40 µM) or CFSE^low^ (4 µM) concentration for 10 min at 37°C. Excess CFSE was quenched with RPMI containing 10% FCS and subsequently washed in medium without FCS. Next, CFSE^high^ labeled cells were pulsed with TB10.4_3-11,_ TB10.4_20-28_, or TB10.4_74-88_ peptides at a concentration of 10 µg/ml for 1.5 hr at 37°C. After being washed and resuspended in PBS the CFSE^high^ and CFSE^low^ suspensions for each peptide was mixed at equal volumes. Finally, 10×10^6^ cells per target peptide per mouse were given intravenously into naïve mice and mice vaccinated one week before. Four hours later, adoptively transferred mice were sacrificed. Spleens were removed, homogenized and resuspended in formaldehyde before acquisition on a BD FACSCanto flowcytometer (BD Biosciences, USA). To evaluate the frequency of specific lysis, the ratio of CFSE^high^ and CFSE^low^ of infected mice were compared to naïve control mice and was calculated using the formula (1−(%CFSE^high^ cells/%CFSE^low^ cells)×100%). For the immunized and naïve groups two mice were used respectively.

## References

[1] Cooper AM, Dalton DK, Stewart TA, Griffin JP, Russell DG (1993). Disseminated tuberculosis in interferon gamma gene-disrupted mice.. J Exp Med.

[2] Dorman SE, Holland SM (2000). Interferon-gamma and interleukin-12 pathway defects and human disease.. Cytokine Growth Factor Rev.

[3] Flynn JL, Chan J, Triebold KJ, Dalton DK, Stewart TA (1993). An essential role for interferon gamma in resistance to Mycobacterium tuberculosis infection.. J Exp Med.

[4] Tascon RE, Stavropoulos E, Lukacs KV, Colston MJ (1998). Protection against Mycobacterium tuberculosis infection by CD8+ T cells requires the production of gamma interferon.. Infect Immun.

[5] Derrick SC, Repique C, Snoy P, Yang AL, Morris S (2004). Immunization with a DNA vaccine cocktail protects mice lacking CD4 cells against an aerogenic infection with Mycobacterium tuberculosis.. Infect Immun.

[6] Turner J, D'Souza CD, Pearl JE, Marietta P, Noel M (2001). CD8- and CD95/95L-dependent mechanisms of resistance in mice with chronic pulmonary tuberculosis.. Am J Respir Cell Mol Biol.

[7] van Pinxteren L, Cassidy JP, Smedegaard BHC, Agger EM, Andersen P (2000). Control of latent *Mycobacterium tuberculosis* infection is dependent on CD8 T cells.. Eur J Immunol.

[8] Muller I, Cobbold SP, Waldmann H, Kaufmann SH (1987). Impaired resistance to Mycobacterium tuberculosis infection after selective in vivo depletion of L3T4+ and Lyt-2+ T cells.. Infect Immun.

[9] Flynn JL, Goldstein MM, Triebold KJ, Koller B, Bloom BR (1992). Major histocompatibility complex class I-restricted T cells are required for resistance to Mycobacterium tuberculosis infection.. Proc Natl Acad Sci USA.

[10] Behar SM, Dascher CC, Grusby MJ, Wang CR, Brenner MB (1999). Susceptibility of mice deficient in CD1D or TAP1 to infection with Mycobacterium tuberculosis.. J Exp Med.

[11] Billeskov R, Vingsbo-Lundberg C, Andersen P, Dietrich J (2007). Induction of CD8 T cells against a novel epitope in TB10.4: correlation with mycobacterial virulence and the presence of a functional region of difference-1.. J Immunol.

[12] Pym AS, Brodin P, Brosch R, Huerre M, Cole ST (2002). Loss of RD1 contributed to the attenuation of the live tuberculosis vaccines Mycobacterium bovis BCG and Mycobacterium microti.. Mol Microbiol.

[13] Pym AS, Brodin P, Majlessi L, Brosch R, Demangel C (2003). Recombinant BCG exporting ESAT-6 confers enhanced protection against tuberculosis.. Nat Med.

[14] Brodin P, Majlessi L, Brosch R, Smith D, Bancroft G (2004). Enhanced protection against tuberculosis by vaccination with recombinant Mycobacterium microti vaccine that induces T cell immunity against region of difference 1 antigens.. J Infect Dis.

[15] McShane H, Pathan AA, Sander CR, Keating SM, Gilbert SC (2004). Recombinant modified vaccinia virus Ankara expressing antigen 85A boosts BCG-primed and naturally acquired antimycobacterial immunity in humans.. Nat Med.

[16] McShane H, Pathan AA, Sander CR, Goonetilleke NP, Fletcher HA (2005). Boosting BCG with MVA85A: the first candidate subunit vaccine for tuberculosis in clinical trials.. Tuberculosis.

[17] Bennekov T, Dietrich J, Rosenkrands I, Stryhn A, Doherty TM (2006). Alteration of epitope recognition pattern in Ag85B and ESAT-6 has a profound influence on vaccine-induced protection against Mycobacterium tuberculosis.. Eur J Immunol.

[18] Radosevic K, Wieland CW, Rodriguez A, Weverling GJ, Mintardjo R (2007). Protective immune responses to a recombinant adenovirus type 35 tuberculosis vaccine in two mouse strains: CD4 and CD8 T-cell epitope mapping and role of gamma interferon.. Infect Immun.

[19] Skjot RL, Oettinger T, Rosenkrands I, Ravn P, Brock I (2000). Comparative evaluation of low-molecular-mass proteins from Mycobacterium tuberculosis identifies members of the ESAT-6 family as immunodominant T-cell antigens.. Infect Immun.

[20] Skjot RL, Brock I, Arend SM, Munk ME, Theisen M (2002). Epitope mapping of the immunodominant antigen TB10.4 and the two homologous proteins TB10.3 and TB12.9, which constitute a subfamily of the esat-6 gene family.. Infect Immun.

[21] Hervas-Stubbs S, Majlessi L, Simsova M, Morova J, Rojas MJ (2006). High Frequency of CD4+ T Cells Specific for the TB10.4 Protein Correlates with Protection against Mycobacterium tuberculosis Infection.. Infect Immun.

[22] Andersen P, Doherty TM (2005). TB subunit vaccines–putting the pieces together.. Microbes Infect.

[23] Dietrich J, Aagaard C, Leah R, Olsen AW, Stryhn A (2005). Exchanging ESAT6 with TB10.4 in an Ag85B fusion molecule-based tuberculosis subunit vaccine: efficient protection and ESAT6-based sensitive monitoring of vaccine efficacy.. J Immunol.

[24] Darrah PA, Patel DT, De Luca PM, Lindsay RW, Davey DF (2007). Multifunctional TH1 cells define a correlate of vaccine-mediated protection against Leishmania major.. Nat Med.

[25] Seder RA, Darrah PA, Roederer M (2008). T-cell quality in memory and protection: implications for vaccine design.. Nat Rev Immunol.

[26] Majlessi L, Rojas MJ, Brodin P, Leclerc C (2003). CD8+-T-cell responses of Mycobacterium-infected mice to a newly identified major histocompatibility complex class I-restricted epitope shared by proteins of the ESAT-6 family.. Infect Immun.

[27] van Pinxteren LA, Cassidy JP, Smedegaard BH, Agger EM, Andersen P (2000). Control of latent Mycobacterium tuberculosis infection is dependent on CD8 T cells.. Eur J Immunol.

[28] Feng CG, Demangel C, Kamath AT, Macdonald M, Britton WJ (2001). Dendritic cells infected with Mycobacterium bovis bacillus Calmette Guerin activate CD8(+) T cells with specificity for a novel mycobacterial epitope.. Int Immunol.

[29] Wang J, Thorson L, Stokes RW, Santosuosso M, Huygen K (2004). Single mucosal, but not parenteral, immunization with recombinant adenoviral-based vaccine provides potent protection from pulmonary tuberculosis.. J Immunol.

[30] McShane H, Behboudi S, Goonetilleke N, Brookes R, Hill AV (2002). Protective immunity against Mycobacterium tuberculosis induced by dendritic cells pulsed with both CD8(+)- and CD4(+)-T-cell epitopes from antigen 85A.. Infect Immun.

[31] McShane H, Brookes R, Gilbert SC, Hill AV (2001). Enhanced Immunogenicity of CD4(+) T-Cell Responses and Protective Efficacy of a DNA-Modified Vaccinia Virus Ankara Prime-Boost Vaccination Regimen for Murine Tuberculosis.. Infect Immun.

[32] Santosuosso M, McCormick S, Zhang X, Zganiacz A, Xing Z (2006). Intranasal boosting with an adenovirus-vectored vaccine markedly enhances protection by parenteral Mycobacterium bovis BCG immunization against pulmonary tuberculosis.. Infect Immun.

[33] Santosuosso M, McCormick S, Roediger E, Zhang X, Zganiacz A (2007). Mucosal luminal manipulation of T cell geography switches on protective efficacy by otherwise ineffective parenteral genetic immunization.. J Immunol.

[34] Novy P, Quigley M, Huang X, Yang Y (2007). CD4 T cells are required for CD8 T cell survival during both primary and memory recall responses.. J Immunol.

[35] Johansen P, Stamou P, Tascon RE, Lowrie DB, Stockinger B (2004). CD4 T cells guarantee optimal competitive fitness of CD8 memory T cells.. Eur J Immunol.

[36] Janssen EM, Lemmens EE, Wolfe T, Christen U, von Herrath MG (2003). CD4+ T cells are required for secondary expansion and memory in CD8+ T lymphocytes.. Nature.

[37] Shedlock DJ, Shen H (2003). Requirement for CD4 T cell help in generating functional CD8 T cell memory.. Science.

[38] Sauzet JP, Gras-Masse H, Guillet JG, Gomard E (1996). Influence of strong CD4 epitope on long-term virus-specific cytotoxic T cell responses induced in vivo with peptides.. Int Immunol.

[39] Thomsen AR, Johansen J, Marker O, Christensen JP (1996). Exhaustion of CTL memory and recrudescence of viremia in lymphocytic choriomeningitis virus-infected MHC class II-deficient mice and B cell-deficient mice.. J Immunol.

[40] Kristensen NN, Christensen JP, Thomsen AR (2002). High numbers of IL-2-producing CD8+ T cells during viral infection: correlation with stable memory development.. J Gen Virol.

[41] Wu Y, Woodworth JS, Shin DS, Morris S, Behar SM (2008). Vaccine-elicited 10-kilodalton culture filtrate protein-specific CD8+ T cells are sufficient to mediate protection against Mycobacterium tuberculosis infection.. Infect Immun.

[42] Becker TC, Noel RJ, Coats WS, Gomez-Foix AM, Alam T (1994). Use of recombinant adenovirus for metabolic engineering of mammalian cells.. Methods Cell Biol.

